# Gastric juice non-coding RNAs as potential biomarkers for gastric cancer

**DOI:** 10.3389/fphys.2023.1179582

**Published:** 2023-04-26

**Authors:** Ilgiz Gareev, Aamir Ahmad, Jiaqi Wang, Aferin Beilerli, Tatiana Ilyasova, Albert Sufianov, Ozal Beylerli

**Affiliations:** ^1^ Educational and Scientific Institute of Neurosurgery, Peoples’ Friendship University of Russia (RUDN University), Moscow, Russian; ^2^ Academic Health System, Hamad Medical Corporation, Interim Translational Research Institute, Doha, Qatar; ^3^ Department of Urology, Harbin Medical University Cancer Hospital, Harbin, China; ^4^ Department of Obstetrics and Gynecology, Tyumen State Medical University, Tyumen, Russia; ^5^ Department of Internal Diseases, Bashkir State Medical University, Ufa, Russia; ^6^ Department of Neurosurgery, Sechenov First Moscow State Medical University (Sechenov University), Moscow, Russia

**Keywords:** gastric juice ncRNAs and gastric cancer non-coding RNAs, gastric cancer, gastric juice, liquid biopsy, non-invasive biomarkers, extracellular vesicles, theories, clinical perspectives

## Abstract

Gastric cancer (GC), being one of the most common malignant human tumors, occupies the second position in the structure of mortality in men and women. High rates of morbidity and mortality in this pathology determine its extremely high clinical and social significance. Diagnosis and timely treatment of precancerous pathology is the main way to reduce morbidity and mortality, and early detection of GC and its adequate treatment improve prognosis. The ability to accurately predict the development of GC and start treatment on time, as well as the ability to determine the stage of the disease if the diagnosis is confirmed - non-invasive biomarkers can become the key to solving these and many other problems of modern medicine. One of the promising biomarkers being studied are non-coding RNAs, namely, miсroRNAs (miRNAs), long non-coding RNAs (lncRNAs), and circular RNAs (circRNAs). They are involved in a wide range of processes, including apoptosis, proliferation, differentiation, angiogenesis, which play a critical role in the development of GC oncogenesis. In addition, they are quite specific and stable due to their carriers (extracellular vesicles or Argonaute 2 protein) and can be detected in various human biological fluids, in particular gastric juice. Thus, miRNAs, lncRNAs, and circRNAs isolated from the gastric juice of GC patients are promising preventive, diagnostic and prognostic non-invasive biomarkers. This review article presents the characteristics of circulating or extracellular miRNAs, lncRNAs, and circRNAs in gastric juice, allowing their use in the GC preventive, diagnosis, prognosis and monitoring therapy.

## 1 Introduction

Gastric cancer (GC) ranks third in the world among malignant neoplasms and second in the structure of oncological mortality after lung cancer. The prognosis depends on the stage of the disease, where the 5-year survival rate of patients with GC is approximately 25%–30%, but this figure for patients with early GC after surgical treatment reaches 95% (Smyth et al., 2020). Unfortunately, more than 40% of all GC are still diagnosed at an advanced stage. Early GC is only 5%–10% of the total number of patients with GC (Petryszyn et al., 2020). In addition, metastases occur in 80%–90% of patients with GC (Petryszyn et al., 2020). As a rule, in the early stages of GC has unexpressed clinical manifestations and non-specific symptoms, which makes it difficult to accurately diagnose and prescribe appropriate therapy.

Diagnosis and timely treatment of precancerous pathology is the main way to reduce morbidity and mortality, and early detection of GC and its adequate treatment improve prognosis. This is facilitated by the timely implementation of such studies as, first of all, gastroscopy, which is the basis for screening and early detection of the disease, methods of ultrasound diagnostics, including endo-ultrasound during gastroscopy, various X-ray methods of research - computed tomography (CT), magnetic resonance imaging (MRI), and fluoroscopy (Yoon and Kim, 2015). But if gastroscopy reveals the very presence of a tumor, then all other studies determine the prevalence of the tumor process, which is a necessary condition for the correct staging of the disease and the choice of optimal tactics, as well as dynamic monitoring of the patient during and after treatment. However, the listed diagnostic methods do not always allow to accurately determine the prevalence of the process before the start of treatment, as, for example, in the case of initial peritoneal carcinomatosis, which is often an intraoperative finding, not to mention the dynamic observation after the treatment (Bleicher and Lambert, 2021). Currently, surgery or endoscopic resection of the gastric mucosa followed by histological analysis of the biomaterial are considered the most accurate diagnostic methods for GC (Yoon and Kim, 2015). One of the new and promising directions in improving the quality of diagnosis and prognosis of tumors is “liquid biopsy” - the determination of tumor biomarkers in human biological fluids using various modern methods of laboratory diagnostics. However, GC biomarkers such as cancer embryonic antigen (CEA), carbohydrate antigens cancer antigen 19–9 (CA19-9), cancer antigen 72-4 (CA72-4) and carbohydrate antigen 50 (CA50) for serum and CEA, CA19-9 and fetal sulfoglycoprotein antigen for gastric juice have rather low specificity and sensitivity and cannot be used for early diagnosis of GC (Wu et al., 2019). An increase in the level of these biomarkers occurs in only 20% of cases of GC and does not have a clear correlation with the stage of the disease at the time of its diagnosis (Matsuoka and Yashiro, 2018; Wu et al., 2019). Thus, there is a need to search for new, more specific and sensitive biomarkers for screening, early diagnosis, and prognosis of GC.

The characterization of the molecular mechanisms of GC development is the key not only to understanding the pathogenesis, but also to the creation of effective diagnostic and therapeutic approaches. Given the heterogeneity of GC, the complex nature of molecular, cellular and clinical information, it is now known that violations of epigenetic mechanisms have a significant impact on the development of GC (Guo and Yan, 2015). At the same time, non-coding RNAs (ncRNAs) are a type of epigenetic regulators of the expression of oncogenes and tumor suppressor genes (Guo and Yan, 2015). NcRNAs refer to RNAs that do not code for protein. They cover a huge number of RNA classes and perform a wide range of biological functions. One such class of ncRNAs that has been extensively studied are microRNAs (miRNAs), long non-coding RNAs (lncRNAs), and circular RNAs (circRNAs) (Guo and Yan, 2015). MiRNAs are endogenous RNAs of 18–22 nucleotides that inhibit gene expression by promoting degradation of their messenger RNA (mRNA) targets or inhibition of translation. It has been proven that miRNAs play an essential role in various biological processes, including the cell cycle, apoptosis, cell proliferation and differentiation, regulating the expression of about one-third of all human genes (Guo and Yan, 2015; Beylerli et al., 2022a).

LncRNAs are a group of ncRNAs longer than 200 nucleotides. Due to their length of nucleotide chains, lncRNAs have the unique ability to take on many complex secondary and tertiary structures, allowing them to perform specific functions such as catalysis, metabolism, and cell differentiation. LncRNAs cannot code for protein, but can modulate gene expression at epigenetic (e.g., DNA methylation and histone modification), transcriptional (e.g., recruitment of transcription factors), and post-transcriptional (e.g., regulation of miRNA and mRNA stability) levels (Guo and Yan, 2015; Ghafouri-Fard et al., 2020; Sufianova et al., 2022). CircRNAs are a class of regulatory ncRNAs with a closed structure of the ribose phosphate backbone. CircRNAs are neither polyadenylated nor capped. They are more stable than linear lncRNAs, making them more promising diagnostic markers and therapeutic agents. Like lncRNAs, circRNAs can interact with other RNAs, DNAs, and proteins and perform various functions in the cell. Many circRNAs contain binding sites for various miRNAs and act as a “sponge”, sorbing these molecules onto themselves (Guo and Yan, 2015; Sekar and Liang, 2019; Beilerli et al., 2022).

An increasing number of studies have demonstrated aberrant expression of a number of miRNAs, lncRNAs, and circRNAs in various types of tumors (Yan and Bu, 2021). The results of some studies have shown that these ncRNAs play a direct role in the development of GC (Xie et al., 2020). In addition, many miRNAs, lncRNAs, and circRNAs are found in the biological fluids of patients with GC: in whole blood, plasma/serum, and gastric juice. NcRNAs are present in the cell, in the cytoplasm, as well as in the nucleus, and are also secreted outside the cell via extracellular vesicles (EVs) (exosomes and microvesicles), which protects them from degradation. Thus, it is already known that the so-called circulating (cell-free or extracellular) miRNAs, lncRNAs and circRNAs are highly stable in biological fluids (including gastric juice), resistant to endogenous ribonucleases and adverse physical conditions, which allows them to be efficiently isolated from biological fluids, where their amount can be measured with high sensitivity and specificity using real-time PCR, DNA microarrays and RNA-sequencing (RNA-seq) methods (Gareev et al., 2020; Beylerli et al., 2022b). Thus, these ncRNAs have great potential for use in clinical practice as non-invasive biomarkers for screening, early diagnosis, prediction, and monitoring of the effectiveness of therapy for patients with GC. In this review paper, we will pay attention to cell-free miRNAs, lncRNAs and circRNAs circulating in the gastric juice, which can be used as biomarkers for minimally invasive diagnosis of GC, prognosis of the course of the disease, and evaluation of the effectiveness of therapy.

## 2 The choice of biological fluid

Since blood vessels permeate all tissues of the body, it is logical to assume that blood is the default source of various biomarkers, including extracellular ncRNAs, but the link between test samples and disease may be more important. So, in diseases of the central nervous system (CNS), you can use cerebrospinal fluid, blood is ideal for cardiovascular diseases, urine - for metabolic diseases, diseases of the liver or kidneys. In diseases of the gastrointestinal tract, including GC, gastric juice or saliva can be used as a source of biomarkers, in diseases of the lungs, saliva, sputum, or even exhaled air vapor (Aronson and Ferner, 2017). For diseases localized in a particular place, tissue or tissue fluid can be used. However, sometimes samples obtained from foci distant from the sources of the disease may also contain suitable biomarkers. There are a significant number of studies in which extracellular miRNAs, lncRNAs and circRNAs have been studied as biomarkers in GC. In all these studies, despite the difference in the choice of ncRNAs and in the search medium (whole blood, serum or plasma and gastric juice), the possibility of using ncRNAs in gastric juice as stable, sufficiently sensitive and specific biomarkers of the development of the tumor process has been shown.

Blood (plasma and serum) is one of the available biological fluids for profiling the expression of circulating ncRNAs in patients with tumors. Blood plasma and serum turned out to be informative for profiling the expression of circulating ncRNAs for the purpose of diagnosing, predicting, assessing response to therapy and tumor recurrence, as well as identifying emerging resistance to therapy in various human tumors, including GC (Jelski and Mroczko, 2022; Mugoni et al., 2022; Powrózek and Ochieng Otieno, 2022). The detection of ncRNAs in plasma samples without measurable circulating tumor cells suggests that circulating miRNAs, lncRNAs and circRNAs can provide useful information about tumors, regardless of the presence of circulating tumor cells.

As described above, EVs are nanoparticles enclosed in membranes that are released from living tumor cells either as a result of fusion of the endosome with the plasma membrane (exosomes) or directly from the cell membrane-microvesicles (Abels and Breakefield, 2016). EVs are carriers of communication between different tumor compartments and its microenvironment, since other tumor cells and normal cells absorb them. It is important to note that EVs, which can be isolated from both blood and gastric juices, are a rich source of oncogenesis molecules such as ncRNA, since the structure of EVs protects them from nucleases, proteases, and the acidic environment of gastric juice (Kagota et al., 2019; Urabe et al., 2020). Unlike blood, gastric juice is in direct contact with the stomach and is a suitable source of biomarkers for gastric cancer. Gastric juice seems to be a very suitable source of EVs for solving the problem of finding non-invasive GC biomarkers, since the expected proportion of EVs originating from tumor cells and cells of the microenvironment should be significantly higher in this body fluid compared to the bloodstream (Skryabin et al., 2022). In addition, unlike blood, gastric juice should not contain ribonucleoproteins and lipoprotein complexes, which almost inevitably contaminate with EV (Skryabin et al., 2022). Surprisingly, gastric juice -derived EVs have barely been explored so far, except for a few studies (Kagota et al., 2019; Zeng et al., 2019; Tanaka et al., 2020; Skryabin et al., 2022). In addition, gastric juice is easier and safer to obtain than tumor tissue from a biopsy. Based on these arguments, this review paper will consider gastric juice miRNAs, lncRNAs and circRNAs in GC. For instance, the Table 1 provides some information on biomarkers and priority biological fluids for a range of tumors (Saijo, 2012; Crawford et al., 2014; Günther, 2015; Hayes, 2015; Sims et al., 2015; Wu and Qu, 2015; Zhou et al., 2015; Usman et al., 2023).

**TABLE 1:** Commonly studied tumor biomarkers from different biological fluids.

Biomarker	Tumor	Application	Whole blood	Serum	Plasma	Urine	CSF	Saliva	Gastic juice	Breast milk	Seminal fluid
Methylation of MGMT promoter	Glioblastoma	Drug response to chemotherapy		X			X				
GlcNAc	Breast cancer	Early detection and risk assessment in young, reproductively active women								X	
CA15-3	Breast cancer	To assess whether treatment is working or if tumor has recurred	X	X	X						
CA27.29	Breast cancer	To detect metastasis or recurrence	X	X	X						
BTA	Bladder cancer	As surveillance with cytology and cystoscopy of patients already known to have bladder cancer				X					
CA-125	Ovarian cancer	To help in diagnosis, assessment of response to treatment, and evaluation of recurrence	X	X	X						
PCA3 mRNA	Prostate cancer	To determine need for repeating biopsy after a negative biopsy				X					
Citrate levels	Prostate cancer	Confirm the proposal that prostate malignancy involves a metabolic transformation									X
ROS1 gene rearrangement	NSCLC	To help determine treatment	X	X	X						
CEA, CA72-4, alpha-fetoprotein, and CA125	Gastic cancer	For early diagnosis and accurate prediction of therapeutic approaches	X	X	X						
α1-antitrypsin	Gastic cancer	Diagnosis of gastric malignancy						X	X		
Ki67 and Cyclin D1	OSCC	For diagnosis, prognosis and post-operative monitoring		X	X						
Thyroglobulin	Thyroid cancer	To evaluate response to treatment and to look for recurrence		X							

**Abbreviations:** NSCLC, non-small cell lung cancer; BTA, bladder tumor antigen; MGMT, O6-methylguanine-DNA, methyl-transferase; GlcNAc, N-acetylglucosamine; PCA3, Prostate cancer antigen 3; CEA, Carcinoembryonic antigen; OSCC, Oral squamous cell carcinoma; CSF, Cerebrospinal fluid; X, detected in sample.

## 3 Extracellular miRNAs, lncRNAs and circRNAs as biomarkers and their advantages

**FIGURE 1:**
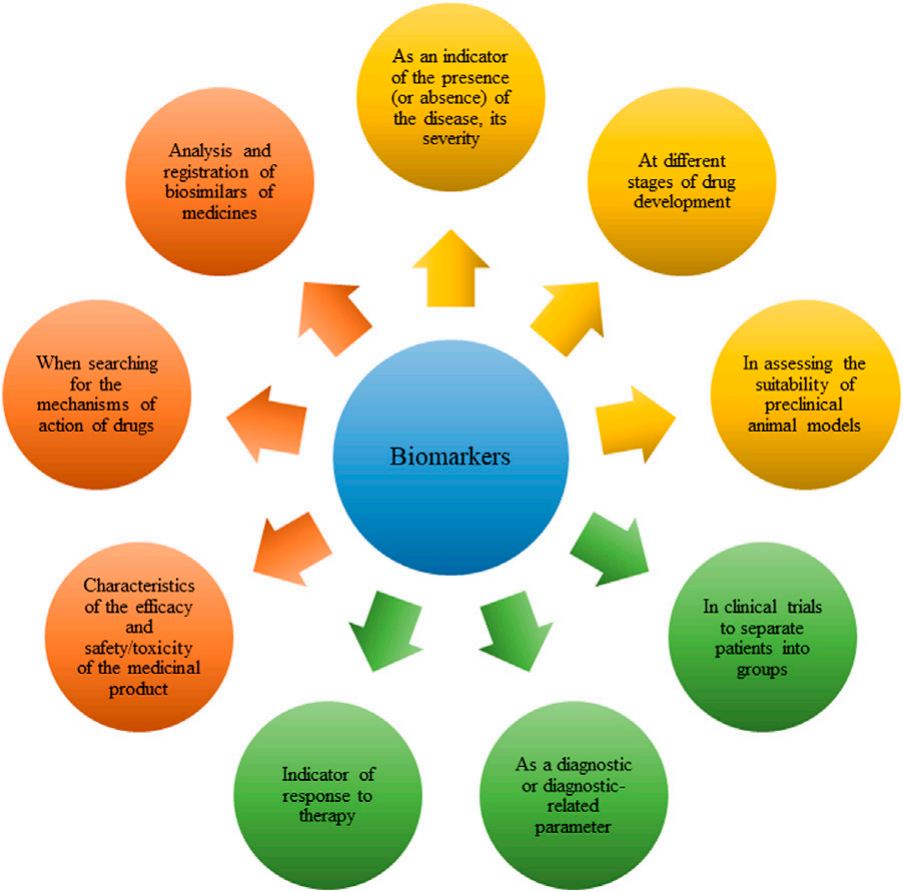
Indications for the use of biomarkers in various diseases, including in gastric cancer (GC).

The ability to accurately predict the development of GC and start treatment on time, the ability to choose therapy depending on the individual characteristics of the patient, the possibility of early diagnosis of the disease and determining the stage - biomarkers can become the key to solving these and many other problems of modern oncology. A biomarker is defined as a quantitatively and objectively measurable indicator of a biological, pathogenic process or pharmacological response to therapy and is used for a number of indicators (Figure 1) (Crowley et al., 2013; Wu and Qu, 2015; Aronson and Ferner, 2017).

**FIGURE 2:**
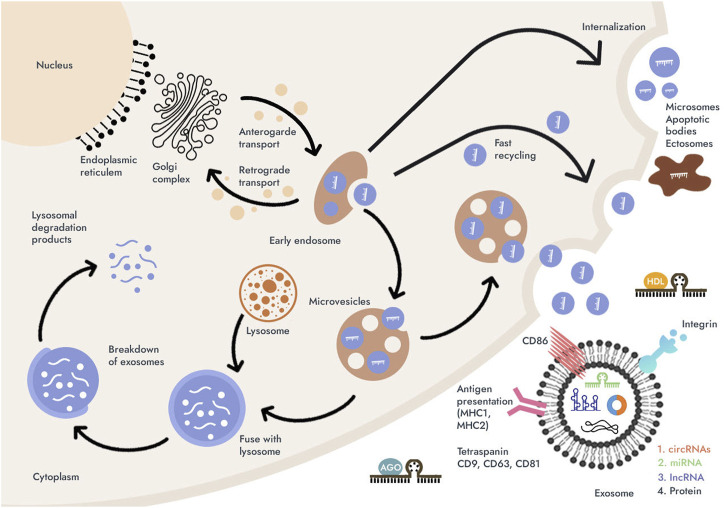
Possible pathways for the secretion of microRNAs (miRNAs), long non-coding RNAs (lncRNAs), and circular RNAs (circRNAs) into the biological fluids (blood or gastric juice) and their vehicles. MiRNAs, lncRNAs, and circRNAs can be secreted by the cell as microscopic extracellular vesicles (EVs) (exosomes and microvesicles) or released as part of apoptotic bodies; miRNAs can be found associated with high-density lipoproteins (HDL) and mostly in the form of complexes with Argonauts proteins (with Ago2). Then, regardless of the forms of miRNAs, lncRNAs and circRNAs pass from the extracellular space into the biological fluid. About 90% of circulating miRNAs are in a non-vesicular form, namely, they are associated with Ago2. In addition to miRNAs, lncRNAs and circRNAs, exosomes contain a diverse array of molecules such as translation factors, metabolic enzymes, apoptotic proteins (Alix), proteolytic proteins chaperones, and contain other types of nucleic acids such as mRNAs. MiRNAs, lncRNAs, and circRNAs account for almost 50% of the total vesicular RNA.

Possessing most of the properties of ideal biomarkers, including resistance to non-nucleases, a unique nucleotide sequence, tissue specificity, minimally invasive and generally available sampling, relative stability at room temperature and repeated freeze/thaw cycles of blood samples, cell-free miRNAs, lncRNAs and circRNAs are deservedly considered in as promising biomarkers. However, to date, a consensus on the origin and biological functions of extracellular miRNAs, lncRNAs and circRNAs has not been formed. This may be due to the fact that the forms of extracellular existence of miRNAs, lncRNAs and circRNAs are inhomogeneous and differ in the way they are packaged. More information is needed on the mechanisms and causes of ncRNAs release from cells, which will be revealed first of all by analyzing the correlations between circulating and tissue concentrations of miRNAs, lncRNAs and circRNAs (Gareev et al., 2020; Szilágyi et al., 2020). However, three secretion pathways for cell-free miRNAs, lncRNAs and circRNAs are currently known: 1) passive secretion from damaged cells due to apoptosis or necrosis; 2) active secretion with the help of explosives, including exosomes and microvesicles, and as part of high density lipoproteins (HDL); and 3) active secretion of circulating miRNAs via an RNA-binding protein-dependent pathway (miRNA- Aurgonaute 2 (Ago2) complex) (Figure 2) (Turchinovich et al., 2011; Ding et al., 2018; Szilágyi et al., 2020; Zhao et al., 2020).

Exosomes are small membrane-bound vesicles of endosomal origin with a diameter of 30–100 nm. They are secreted by different types of cells: normal or pathological. Previously, exosomes for the cell were considered as a way to get rid of unnecessary “garbage”, for example, “obsolete” proteins. However, it has now been proven that exosomes are more than just “garbage bins” and take a significant part in intercellular communication (Kalluri and LeBleu, 2020). Exosomes, like microvesicles, are used by cells to transfer information (mRNA, viruses, proteins, miRNAs, lncRNAs and circRNAs) from 1 cell to another. Tumor cells play a particularly important role in the production of exosomes. Exosomes of tumor cells are able to remotely participate in the formation of “niches” (Zhang and Yu, 2019). In addition to vesicular forms of extracellular miRNAs, long noncoding RNAs, and circular RNAs, apoptotic bodies or cell masses 1–4 nm in size after necrotic processes, which also contain ncRNA data, can be found in biological fluids cell-free miRNAs, lncRNAs and circRNAs can also be secreted outside the vesicles. About 90% of circulating miRNAs are in a non-vesicular form, namely, they are associated with Ago2 proteins. It is reported that HDL are involved in the mechanism of intercellular communication and are involved in the transport and delivery of ncRNA (Turchinovich et al., 2011; Sims et al., 2015; Xu et al., 2022). However, the origin of the lipoprotein fraction of circulating miRNAs, lncRNAs and circRNAs is still in question.

### 3.1 Preventive (screening) diagnostic

There is no evidence for the efficacy of screening for GC based on randomized controlled trials. The only country that screens for stomach cancer is Japan, due to the high incidence of this form of cancer. The survey increases the early diagnosis of GC by 30%–40% (Mabe et al., 2022). In European countries, screening is not practiced because of the high cost, in the United States - because of the low incidence. In the United Kingdom, only patients with chronic stomach conditions are screened (Crew and Neugut, 2006). Early diagnosis of gastric cancer leads to more effective therapy, resulting in an increase in the duration and quality of life of patients. Therefore, the search for reliable and accurate ways to predict the malignancy of normal stomach cells into cancer cells is an acute problem to this day.

As already mentioned, the use of liquid biopsy based on the detection of specific molecules in human biological fluids for malignancy processes is an important direction in the field of preventive diagnosis of GC. Protein molecules as biomarkers are sensitive indicators of normal biological, pathogenic processes, including oncogenesis, or response to a specific intervention or therapy. Although circulating CEA, CA19.9, and CA72.4 have notable predictive value for predicting GC recurrence and metastases after surgery and chemotherapy, as of 2017, none of these tumor antigens had significant value for gastric screening (Wu et al., 2019; de Mello et al., 2021). In particular, circulating ncRNAs in plasma or serum have been found to be extremely stable after long-term storage or repeated freeze-thaw cycles and can be reliably studied in both fresh and stored samples (Vogiatzi et al., 2018). The main reasons for this stability are their associations with protein complexes or small membrane vesicles known as exosomes or microvesicles (Gareev et al., 2020).

In addition, circulating miRNAs, lncRNAs and circRNAs have advantages over protein-based biomarkers because they are less complex, are found in different tissues or biological media, and can be easily identified using common laboratory methods. Circulating miRNAs, lncRNAs and circRNAs can be detected at the early stages of oncogenesis, when protein tumor markers are already active at the active stage of tumor development and progression. That is, aberrant expression of extracellular ncRNAs indicates the disease even before the manifestation of the clinical picture (in the latent period), and the profile may vary depending on the degree and severity of the disease, which is especially important for determining the stage of oncological disease (Schwarzenbach et al., 2011; Sims et al., 2015; Diez-Fraile et al., 2020; Gareev et al., 2020). All this makes them potentially useful as non-invasive biomarkers for use in screening studies for GC.

### 3.2 Diagnostic value

One of the main factors determining the tactics of managing patients with primary or metastatic GC is the determination of the type of tumor. The type of GC is usually identified on the basis of studies of the tumor tissue (biopsy) performed during gastroscopy, and the subsequent decision on the type of treatment depends on the results of the biopsy (Yoon and Kim, 2015). Even for those patients who are suspected to have benign gastric tumors, tissue biopsy is currently required to establish an accurate diagnosis and plan further care. The ability to distinguish benign gastric tumors from GC in real time by analyzing biomarkers based on gastric juice will help plan the course of treatment (e.g., use neoadjuvant therapy). In addition, in the modern age of using molecular parameters in the classification of GC, certain circulating ncRNAs as biomarkers with predictive value can help in planning operations, decision making during surgery and enrollment in clinical trials (Choi et al., 2019).

### 3.3 Detection of recurrence

Monitoring the risk of recurrence in the immediate or long term after surgical, radio- and chemotherapeutic treatment of GC is a difficult task (Foller et al., 2018). Due to the specialist’s inability to distinguish pseudoprogression from recurrence using imaging studies, a number of patients without true GC recurrence undergo potentially unnecessary surgical procedures, such as repeat tissue biopsy, to confirm tumor recurrence, which (surgical manipulation) may have a high sampling error rate. It is assumed that changes in the expression level of circulating ncRNAs can correlate with tumor burden. In this scenario, it is reasonable to assume that the progression of GC will lead to an increase in the level of expression of certain circulating miRNAs, lncRNAs and circRNAs in GC patients, and this will help to identify the progression already at the molecular level (Zhang et al., 2019a).

### 3.4 Monitoring response to therapy

Of the instrumental methods for examining GC, X-ray and endoscopic methods are mainly used, where these methods often do not provide convincing evidence that allows one to observe the effectiveness of the response to tumor treatment (Yoon and Kim, 2015). As mentioned earlier, CEA, CA19.9, and CA72.4 are biomarkers for several tumors, including GC, which are found in plasma or serum; however, none of them was effective in monitoring the quality of therapy (de Mello et al., 2021). The lack of sensitive and specific biomarkers to assess response to treatment in gastric cancer patients makes it difficult to develop new therapeutic agents (Wu and Qu, 2015). Future studies should aim to fill this knowledge gap by systematically evaluating gastric juice samples for circulating ncRNAs and correlating the expression level of specific circulating ncRNAs with tumor volume as determined by radiological and endoscopic techniques.

## 4 Gastric juice miRNAs and GC

Analysis of the expression of extracellular miRNAs in gastric juice can face a number of problems. Gastric juice is a mixture consisting mainly of proteins and hydrochloride (HCl). Chen et al. demonstrated in their work that microRNAs (e.g., miR-25, miR-223, and let-7a) of serum and plasma treated for 3 h with exogenous HCl solution remain stable. In doing so, they examined the stability of circulating miRNAs under conditions of boiling, low/high pH, and freeze-thaw cycles; this indicates that miRNAs in gastric juice are stable and can be used as new biomarkers for GC (Chen et al., 2008). In another study, Cui et al. observed that the real-time quantitative reverse transcription–polymerase chain reaction (qRT-PCR) method for detecting extracellular miRNAs in gastric juice was satisfactorily reproducible, and sequencing results confirmed the stability of miRNAs in gastric juice (Cui et al., 2013). In addition, they observed significantly lower expression levels of circulating miR-21 and miR-106a in the gastric juice of patients with GC compared to the gastric juice of patients with benign gastric diseases. However, expression levels of circulating miR-21 and miR-106a have been reported to be elevated in the plasma and tissues of GC patients, as confirmed by gastric mucosal biopsy. The results of this study are indeed noteworthy. Therefore, in the continuation of this study, the authors found that circulating miR-21 and miR-106a based on gastric juice have a more reliable diagnostic value compared to circulating miR-21 and miR-106a based on blood, where the receiver operating characteristic (AUC) reached 0.96 and 0.87, respectively. Shao et al. also demonstrated that cell-free miR-133a expression levels in gastric juice were significantly lower in GC patients than in healthy individuals and those with benign gastric disease, in particular demonstrating superior diagnostic value, namely, with an AUC of 0.90 and a sensitivity of 85 .9% and a specificity of 84.8%, respectively (Shao et al., 2016).

Several other studies have also shown aberrant expression of a number of other circulating miRNAs, such as miR-129-1-3p, miR-129-2-3p and miR-421, in gastric juice, which may also be good candidates as non-invasive biomarkers in GC (Zhang et al., 2012; Yu et al., 2013). After all, there is evidence that some of these circulating miRNAs, like miR-421, are involved in oncogenic activity, and the authors found them in low amounts in GC patients (Ge et al., 2016; Yang et al., 2017; Jingyue et al., 2019). There are suggestions that these miRNAs may be involved in the process of crosstalk in the tumor microenvironment by being secreted into the gastric juice. All authors who have studied gastric juice miRNAs have come to the conclusion that clinicians can easily obtain samples of gastric juice using a string test that is both economical and acceptable to patients. Thus, for the diagnosis of GC, the use of circulating miRNAs in gastric juice has obvious advantages, since gastric juice is present in direct contact with GC. Table 2 summarizes the results of studies demonstrating the potential of gastric juice miRNAs as non-invasive biomarkers in GC (Zhang et al., 2012; Cui et al., 2013; Yu et al., 2013; Shao et al., 2016; Skryabin et al., 2022)

**TABLE 2:** Summarized several microRNAs (miRNAs) presenting modified circulating expression in gastric juice of gastric cancer (GC) patients compared to healthy controls or benign gastric diseases.

MiRNA	Regulation	Number of GC patients, n	AUC	Sensitivity, %	Specificity, %	Conclusion of study	References
miRNA-21	Down	15	0.969	85.7	97.8	Could be used as early biomarkers of GC	Cui et al. (2013)
miR-106a	Down		0.871	73.8	89.3		
miR-133a	Down	62	0.907	85.9	84.8	Gastric juice miR-133a has the characteristic of high operability, high reliability, high sensitivity, high specificity and relative stability, and suitable for GC screening	Shao et al. (2016)
miR-129-1-3p	Down	42	0.639 0.651 0.656 (combination)	0.452 0.429 0.687 (combination)	0.838 0.859 0.719 (combination)	These gastric juice miRNAs may be a new choice for the diagnosis of GC	Yu et al. (2013)
miR-129-2-3p	Down						
miR-421	Down	42	0.767	71.4	71.7	Gastric juice miR-421 is useful biomarker for screening GC	Zhang et al. (2012)
Exosomal miR-135b-3p and miR-199a-3p	Up Down	7	-	-	-	Exosomes derived from gastric juice are a promising source of	Skryabin et al. (2022)
Exosomal miR-451a			-	-	-	miRNA markers of GC. Participation in the oncogenesis of GC	

**Abbreviations:** AUC, Area Under Curve; -, Not mentioned.

## 5 Gastric juice lncRNAs and GC

LncRNAs are an important group of ncRNAs ranging in length from several hundred to several thousand nucleotides. Genetic changes and aberrant lncRNA expression may be a factor in the development of cancer. Several lncRNAs, such as H19 and LINC00152, correlate with progression and metastases of GC (Li et al., 2014; Wang et al., 2021). Li et al. demonstrated that H19 was significantly increased in GC tissues than in noncancerous tissues, and H19 expression was positively correlated with lymph node metastases (LNM) and clinical stage (Li et al., 2014). In addition, the oncogenic effect of H19 in GC is mediated by direct activation of ISM1 and indirect downregulation of CALN1 expression via miP-675. In other study, Wang et al., demonstrated that LINC00152 expression is upregulated in GC and positively correlated with GC progression (Wang et al., 2021). Moreover, LINC00152 knockdown plays an anti-tumor role in GC by targeting miR-138/SIRT2 axis.

Cell-free lncRNAs have great potential to be considered as non-invasive biomarkers. Because they are more stable, have high tissue/cell specificity, and are readily detectable in body fluids (e.g., gastric juice) (Szilágyi et al., 2020). In addition, there is increasing evidence that aberrant expression of circulating lncRNAs is of clinical importance in the diagnosis and prognosis of GC. As a new diagnostic and prognostic non-invasive biomarker in gastric juice of GC patients, circulating H19, LINC00152 and others are already being studied. For instance, Chen et al. showed that H19 expression was significantly higher in GC tissues compared with adjacent normal tissues (Chen et al., 2016). Moreover, the authors demonstrated that the high H19 group GC patients showed higher invasion depth, advanced TNM (tumor, nodus and metastases) stage and regional lymph nodes metastases than the lower H19 expression group GC patients. In this study, GC patients with a high expression of H19 seemed to have shorter overall survival (OS) and disease-free survival (DFS) than GC patients with lower levels. In addition, the authors found that H19 levels in gastric juice from patients with GC were significantly higher than those from normal subjects. In this study, the authors suggested that H19 might be useful as a diagnostic and prognostic biomarker for GC and might be a possible will be as non-invasive biomarker based gastric juice. However, further studies will be needed to confirm these preliminary results. Pang et al., showed that the expression level of LINC00152 in GC tissues was significantly higher than that in matched paracancerous tissues (Pang et al., 2014). Increased expression levels of LINC00152 was significantly associated with the depth of GC invasion. In addition, the results showed that cell-free LINC00152 levels in gastric juice from GC patients were significantly higher than those from normal controls. Circulating lncRNAs that have been linked to gastric juice in GC are listed in Table 3 (Pang et al., 2014; Shao et al., 2014; Chen et al., 2016; Fei et al., 2016; Yang et al., 2016; Yuan et al., 2016).

**TABLE 3:** Summarized several long non-coding RNAs (lncRNAs) presenting modified circulating expression in gastric juice of gastric cancer (GC) patients compared to healthy controls or benign gastric diseases.

LncRNA	Regulation	Number of GC patients, n	AUC	Sensitivity, %	Specificity, %	Conclusion of study	References
H19	Up	128	-	-	-	H19 might be useful as a non-invasive biomarker for GC	Chen et al. (2016)
LINC00152	Up	106	-	-	-	LINC00152 might be useful as a non-invasive biomarker for GC	Pang et al. (2014)
PVT1	Up	111	-	-	-	PVT1 might be useful as a non-invasive biomarker for GC	Yuan et al. (2016)
AA174084	Up	130	-	-	-	Positive detection rate for early GC was up to 57.1%. Gastric juice AA174084 levels were associated with tumor size, tumor stage, Lauren type, and gastric juice CEA levels. Gastric juice AA174084 as non-invasive biomarker for early diagnosis and for prognosis evaluation	Shao et al. (2014)
ABHD11-AS1	Up	39	0.653	41.0	93.4	Positive detection rate for early GC was up to 71.4%. Gastric juice ABHD11-AS1 levels were with gender, tumor size, tumor stage, Lauren type, and blood CEA levels. Gastric juice ABHD11-AS1 as non-invasive biomarker for early diagnosis and for prognosis evaluation	Yang et al. (2016)
LINC00982	Up	27	-	-	-	LINC00982 might be useful as a non-invasive biomarker for GC	Fei et al. (2016)

**Abbreviations:** AUC, Area Under Curve; PVT1, Plasmacytoma variant translocation 1; ABHD11-AS1, ABHD11 antisense RNA, 1; CEA, Carcinoembryonic antigen; -, Not mentioned.

## 6 Gastric juice circRNAs and GC

Thanks to the achievements of bioinformatics and RNA-seq methods, it can be argued that the circRNAs deserve recognition of the “new players” in the regulation of genes expression. However, the functional significance of the circRNAs is still far from understanding compared to progress achieved in the field of other ncRNAs such as miRNAs and lncRNAs. The vast majority of the circRNAs has not yet been studied, and their functions remain unknown. In addition, in a number of works it is reported that the same circRNAs are involved in various processes of GC progression (Shan et al., 2019). Secondly, the strategies that can be used to deeply study the gastric juice circRNAs for GC are not sufficiently developed. For instance, Shao et al., used microarray method to investigate the differential expression profiles of circRNAs between GC tissues and paired noncancerous tissues (Shao et al., 2017). They identified 20 top differentially expressed circRNAs in GC tissue, where ten upregulated were hsa_circ_0035445, hsa_circ_0003789, hsa_circ_0063809, hsa_circ_0074362, hsa_circ_0006282, hsa_circ_0011107, hsa_circ_0084606, hsa_circ_0005556, hsa_circ_0050547, and hsa_circ_0006470, while the top ten downregulated ones were hsa_circ_0007099, hsa_circ_0001897, hsa_circ_0007707, hsa_circ_0008832, hsa_circ_0001546, hsa_circ_0002089, hsa_circ_0004680, hsa_circ_0000154, hsa_circ_0004458, and hsa_circ_0008394. Further, using the qRT-PCR, the authors identified the specific gastric juice hsa_circ_0014717could be used as a biomarker for screening GC patients. In results, they showed that cell-free hsa_circ 0014717 can stably exist in human gastric juice, and cell-free hsa_circ 0014717 has the potential to be used as biomarkers for the screening of people with high-risk of GC. In other study, almost the same team led by Shao Y. detected and compared gastric juice hsa_circ_0065149 levels in healthy volunteers, gastric ulcer patients, chronic atrophic gastritis patients, and GC patients (Shao et al., 2020). Unexpectedly, maybe due to small number of samples tested, hsa_circ_0065149 levels have no significant difference among four groups. However, the expression levels of exosomal hsa_circ_0065149 in plasma from patents with early GC were significantly decreased than those from healthy control. Moreover, it should be noted that the levels of exosomal hsa_circ_0065149 in gastric juice do not correspond to its levels in GC tissues and levels of exosomal hsa_circ_0065149 in plasma. The authors conclude that this opposite trend may be related to the function of exosomes; and the mechanism of selective change hsa_circ_0065149 in various body fluids needs further study. Song et al., showed that the hsa_circ_000780 levels were significantly decreased in the gastric juice of the GC group patients. However, no significant difference in gastric juice hsa_circ_000780 levels was found between the advanced GC and early GC groups or between the chronic non-atrophic gastritis and chronic atrophic gastritis groups (Song et al., 2021). This finding indicates that cell-free hsa_circ_000780 could be detected in the gastric juice, and has the potential for use as a non-invasive biomarker for early GC screening.

Regarding the use of extracellular circRNAs based on gastric juice as non-invasive biomarkers GC, we come to the conclusion that this area of study is still in its infancy. The studies are represented by a small sample size, the absence of independent comparison groups and a limited analysis of the correlation with the characteristics of the disease.

## 7 Potential non-invasive biomarkers

**FIGURE 3:**
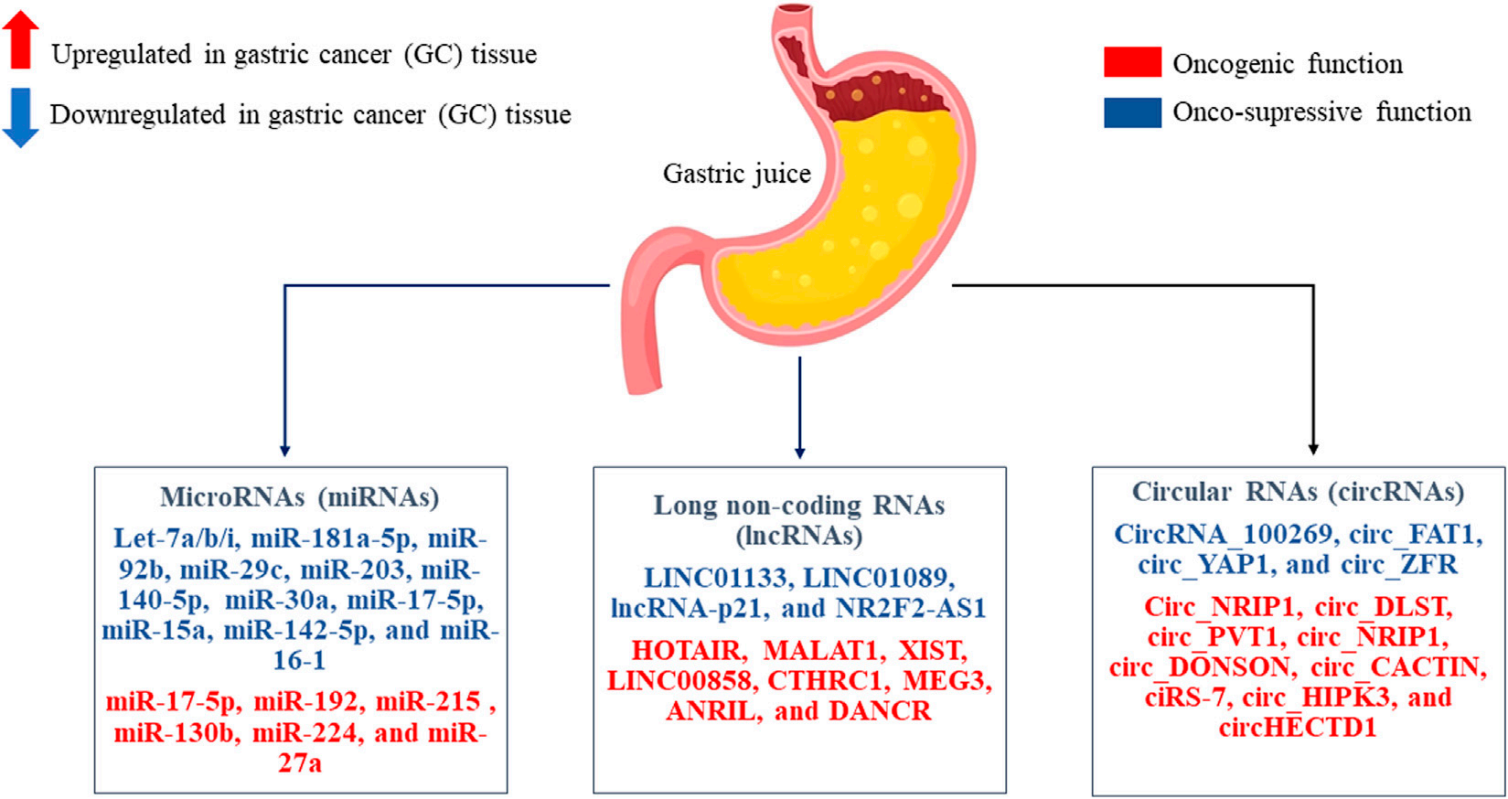
The most studied/studied microRNAs (miRNAs), long non-coding RNAs (lncRNAs), and circular RNAs (circRNAs) in the oncogenesis of gastric cancer (GC) tissue are presented. These non-coding RNAs (ncRNAs) that play a role as onco-suppressors or oncogenic role. Perhaps it will be possible to consider them as non-invasive biomarkers based on gastric juice in GC with further study.

Gastric juice can be a good source of circulating ncRNAs as biomarkers for GC, since it is directly secreted by cells without elimination by the liver. Nevertheless, today there are not so many papers that have studied circulating ncRNAs as non-invasive biomarkers based on gastric juice with GC. Therefore, there is a reason to consider the most studied/studied miRNAs, lncRNAs, and circRNAs which directly interpret in the oncogenesis of GC, and which can be directly considered in future studies on the study of non-invasive biomarkers based on gastric juice for GC screening, diagnosis and prognosis (Figure 3).

Tumor-suppressive let-7 family is generally downregulated in GC tissues and inhibit GC formation or metastases by attenuating some oncogenes. For instance, let-7a expression is significantly reduced in GC tissues, and its onco-suppressive function is associated with inhibition of GC cell differentiation. In addition, upregulation of let-7a leads to inhibition of GC cell proliferation and GC growth due to a decrease in the expression level of RAB40C (Yang et al., 2011). In addition, let-7a is able to induce cellular autophagy by inhibiting rapamycin-insensitive companion of mammalian target of rapamycin (RICTOR) expression in GC cells (Fan et al., 2018). Another member of the let-7 family, let-7b is also downregulated in GC tissues, and its suppression is negatively correlated with poor survival and LNM in GC patients (Motoyama et al., 2008). Overexpression of let-7b suppresses the invasion and migration of GC cells by reducing the expression of inhibitor of growth protein 1 (ING1) (Han et al., 2015). Let-7b also attenuates GC growth and resistance of GC cells to cisplatin by inhibiting Aurora kinase B (AURKB) (Han et al., 2018). In addition, let-7i acts as a tumor suppressor, preventing invasion and metastases of GC cells, promoting the inactivation of collagen, type I, alpha 1 (COL1A1) (Shi et al., 2019).

HOX antisense intergenic RNA (HOTAIR) is known to be an oncogenic lncRNA in GC. Increased HOTAIR expression is highly correlated with LNM, TNM staging, and poor overall survival in patients with GC (Liu et al., 2016). HOTAIR enhances the invasiveness and process of epithelial-mesenchymal transition (EMT) of GC cells, as well as the expression of matrix metallopeptidase-1 (MMP-1) and matrix metallopeptidase-3 (MMP-3) (Xu et al., 2013). Or metastases-associated lung adenocarcinoma transcript 1 (MALAT1) is also oncogenic lncRNAs. It is known that its overexpression correlates with a poor prognosis in patients with GC (Xu et al., 2021). MALAT1 is involved in the direct progression and metastases of GC by activating E-cadherin/β-catenin, extracellular signal-regulated kinases (ERK)/MMP and focal adhesion kinase (FAK)/paxillin signaling pathways (Li et al., 2017).

CircNRIP1 and circDLST, whose expression is elevated in GC tissues, are termed as oncogenic circRNAs, which promote GC progression and metastases. A decrease in circDLST expression inhibits cell viability, invasion, and liver metastases of GC (Chen et al., 2020). CircDLST has been shown to activate the neuroblastoma RAS viral (v-ras) oncogene homolog (NRAS)/MEK1/Ras-dependent extracellular signal-regulated kinase 1/2 (ERK 1/2) signaling pathway, acting as a miR-502–3p sponge in GC cells (Zhang et al., 2019b). Although the function of the circDLST/miR-502–3p axis in the progression of GC has been determined, evidence for the presence of the NRAS/MEK1/ERK1/2 signaling pathway in this role is still insufficient. In a recent study, it was found that circNRIP1 is significantly upregulated in human GC tissues and can successfully inactivate the onco-suppressive miR-149–5p, promoting proliferation, migration, and invasion of GC cells (Zhang et al., 2019a). In addition, inhibition of circNRIP1 expression can block the malignant behavior of GC cells through a RAC-alpha serine/threonine-protein kinase 1 (AKT1)/mammalian target of rapamycin (mTOR) signaling pathway.

## 8 Author opinions

As can be seen from this work of the currently available literature, liquid biopsy is a promising direction in the diagnosis and dynamic monitoring of cancer patients. In the case of GC, when histological material sampling can be performed during routine studies (gastroscopy), it is most likely that liquid biopsy for primary diagnosis is unlikely to find its distribution. Although, if a highly sensitive and specific biomarker common to a particular nosology is detected, as in the case of ncRNAs, it can be used for screening in the early stages of GC, which may be more convenient for the patient than other diagnostic methods.

However, the main application of liquid biopsy is seen in predicting the aggressiveness of the tumor process. For example, when aberrant miRNAs, lncRNAs or circRNAs expression in tissue or biofluids is detected, it may indicate high GC aggression, even in the absence of distant metastases. Thus, dynamic monitoring of patients with gastric cancer becomes more effective if a relapse of the disease is suspected much earlier than it can be detected by modern instrumental research methods, which will allow timely prescribing or changing the necessary therapy. It is possible to control the effectiveness of chemotherapy treatment, both conventional regimens and targeted therapy, which would allow changing the drug or treatment regimen at the right time.

Applicable to GC, the detection of extracellular ncRNAs could play an important role in the dynamic monitoring of patients undergoing radical surgery, due to the rather aggressive course of this disease even at its early stages, the high frequency of relapses and progression, and the low survival rate of these patients. For example, it is known that the “gold standard” for screening and diagnosing prostate cancer (PC) is the determination of the level of prostate-specific antigen (PSA). However, PSA is not a tumor-specific marker, but an organ-specific one: an increase in PSA is caused not only by PC, but also by benign prostatic hyperplasia and chronic prostatitis. Prostate cancer specific antigen 3 (PCA3) is a lncRNA that is specific to the prostate. Increased expression of PCA3 is noted in prostate cancer (in 95% of all cases of PC) (Gareev et al., 2021). PCA3 expression is superior to total PSA and percent free PSA in determining PC in patients with elevated PSA, as PCA3 has a significantly higher AUC (Gareev et al., 2021). PCA3 testing is most useful in determining the need for repeat biopsy in patients with negative histological findings from primary biopsy specimens. In other words, the PSA-3 test helps to detect PC already in routine clinical practice at different stages - from early to progressive. This confirms the future potential of using ncRNA in clinical practice for GC patients.

One of the key issues in the field of research on extracellular ncRNAs as non-invasive biomarkers is the choice of priority biological fluid (whole blood, plasma or serum, and gastric juice), and it must be addressed in order to maximize the potential of circulating miRNAs, lncRNAs and circRNAs for prevention (screening), real-time diagnostics, prognosis and tracking the effectiveness of therapy in GC patients. Venipuncture is a minimally invasive, simple and affordable method for obtaining biomaterial, and therefore the study and detection of biomarkers in the blood are of great interest. However, gastric juice obtained by gastroscopy is in direct contact with tumor tissue, and we suggest that circulating ncRNAs obtained from it can serve as more reliable biomarkers of GC. In addition, the change in the expression profile of circulating miRNAs, lncRNAs and circRNAs in gastric juice and blood in the same GC patient is unique, since gastric juice probably reflects local events of damaged gastric mucosa compared to blood flow. In addition, a specific panel of circulating ncRNAs in gastric juice can help distinguish GC from precancerous diseases of the gastric, which can be indispensable during screening examinations.

Nevertheless, despite the availability of various modern and sufficiently accurate methods of liquid biopsy, only a few are used in clinical practice and for a limited number of pathologies. Perhaps this is due to a large number of techniques, the lack of common markers for a specific pathology, the complexity of performing research and the economic component of the issue. Therefore, new methods for determining circulating miRNAs, lncRNAs and circRNAs in gastric juice should be developed, and the targets for their search should be standardized for more sensitive and specific diagnostics, which will undoubtedly be carried out in the near future due to the great promise in GC.

## 9 Conclusion

It is no longer a secret that miRNAs, lncRNAs and circRNAs are involved not only in physiological processes in the human body, but also interacting with each other, participate in the proliferation of tumor cells, in the control of the cell cycle and apoptosis, migration, invasion and angiogenesis, affecting numerous target genes. These ncRNAs are also involved in the regulation of malignancy and differentiation of normal gastric cells into cancer cells, thus showing that the aberrant expression of some miRNAs, lncRNAs and circRNAs correlates with the clinical prognosis of GC. The use of gastric juice miRNAs, lncRNAs and circRNAs as biomarkers for GC will be ideal when methods for profiling the expression of circulating ncRNAs become sufficiently sensitive and specific for quantification with small sample volumes. Despite advances in expression profiling technology for gastric juice miRNAs, lncRNAs and circRNAs, their use as biomarkers has some limitations. One of them is the lack of proper protocols for handling and storing specimens in the clinical setting. Limited knowledge of environmental factors that may influence the expression of circulating miRNAs, lncRNAs and circRNAs in GC patients may also limit clinical use.
